# Association Between Succinylated Gelatin Exposure and Acute Kidney Injury in Critically Ill Trauma Patients: A Retrospective Five-Year Cohort Study

**DOI:** 10.3390/jcm15218598

**Published:** 2026-11-04

**Authors:** Benjamin Treichl, Mirjam Bachler, Jennifer Gebetsberger, Philipp Martin Lichtenberger, Pia Tscholl, Johannes Bösch, Daniel Niederbrunner, Daniel Stürzel, Michael Rauscher, Dietmar Fries

**Affiliations:** 1 Department of Anaesthesia and Intensive Care Medicine, Medical University Innsbruck, 6020 Innsbruck, Austria; benjamin.treichl@i-med.ac.at (B.T.); daniel.stuerzel@tirol-kliniken.at (D.S.);; 2 Division of Intensive Care and Emergency Medicine, Department of Internal Medicine, Medical University of Innsbruck, 6020 Innsbruck, Austria; 3 Institute for Sports Medicine, Alpine Medicine and Health Tourism, UMIT–University for Health Sciences, Medical Informatics and Technology, 6060 Hall in Tirol, Austria; jennifer.gebetsberger@i-med.ac.at; 4 Data Lab Hell, Non-University Research Institution, 6170 Zirl, Austria

**Keywords:** acute kidney injury, colloids, gelatin, critical illness, fluid resuscitation, critical care

## Abstract

**Background/Objectives:** Early fluid resuscitation is a cornerstone of trauma care, but the relationship between resuscitation strategies and acute kidney injury (AKI) remains insufficiently characterized. This study aimed to evaluate whether administration of 4% succinylated gelatin is associated with the development of AKI in critically ill trauma patients during the first seven days of intensive care unit (ICU) treatment. **Methods:** We conducted an observational retrospective single-center cohort study including 215 adult trauma patients admitted to the ICU at a tertiary care center between 2015 and 2020. Gelatin exposure, kidney function, and clinical outcomes were assessed from the pre-ICU phase through the first ICU week. The primary outcome was the development of AKI, at stage ≥ 2 of the Kidney Disease: Improving Global Outcomes (KDIGO) staging. We used mixed-effects models, competing-risk analyses, correlation analyses and exploratory unsupervised clustering to explore associations between gelatin use and renal outcomes. **Results:** AKI occurred in 17% of patients and was associated with older age, higher Simplified Acute Physiology Score 3 (SAPS3), pre-existing comorbidities, and early signs of hemodynamic and metabolic compromise. Gelatin doses of ≥10 mL/kg/day were not associated with a consistent rise in serum creatinine or progression of AKI over time. Multivariable regression models revealed no independent association between gelatin and worsening AKI. Instead, KDIGO stage worsening was primarily associated with AKI present at ICU admission and with crystalloid volume. Clustering analysis identified a high-risk phenotype with increased gelatin exposure, but this cluster also showed pronounced shock and inflammation, suggesting confounding by indication. In a competing-risk analysis, gelatin was not associated with new-onset AKI, whereas higher gelatin doses were associated with a lower rate of ICU discharge. **Conclusions:** In this retrospective cohort of critically ill trauma patients, succinylated gelatin administration showed no statistically significant association with KDIGO-defined AKI progression across unadjusted and adjusted models. Gelatin doses of ≥10 mL/kg/day were more frequently administered in patients with greater hemodynamic instability, suggesting that gelatin use primarily reflected underlying illness severity rather than acting as a direct nephrotoxic agent. These findings provide no evidence of an independent nephrotoxic effect of gelatin when used as part of balanced trauma resuscitation strategies.

## 1. Introduction

Early fluid resuscitation is a cornerstone of initial trauma management, aiming to restore intravascular volume and maintain organ perfusion in patients with hemorrhagic shock [1,2,3].

Acute kidney injury (AKI) is a frequent complication in critically injured patients, affecting approximately 10–15% of trauma intensive care unit (ICU) admissions and markedly increasing mortality risk. Consequently, minimizing potentially iatrogenic contributors to kidney injury, including the choice of resuscitation fluids, has become an important focus of contemporary trauma care.

In hemorrhagic shock, AKI is largely driven by renal hypoperfusion leading to a reduced glomerular filtration rate, which may progress to ischemic tubular injury if circulatory compromise persists. Beyond acute hemodynamic compromise, pre-existing metabolic and renal risk factors may further influence individual susceptibility to kidney dysfunction [4,5]. Crystalloids remain first-line therapy for hypovolemia, yet colloid solutions have historically been used due to their greater intravascular persistence and lower volume requirements for plasma expansion [6].

Several clinical studies such as the GENIUS, ICARUS-ED or PHOENICS trials have reported improved short-term hemodynamic stabilization with colloid solutions compared to crystalloids, although safety concerns remain central to clinical decision-making [7,8,9].

Among synthetic colloids, hydroxyethyl starch (HES) was widely used in critical care until large trials demonstrated increased risks of AKI, coagulopathy, and mortality in septic and critically ill patients [6]. These findings prompted major regulatory interventions, and in 2013 the European Medicines Agency (EMA) and others restricted the use of HES to non-critically ill trauma patients with acute blood loss, contraindicating its use in septic, burn, or critically ill populations [10]. As subsequent audits revealed ongoing off-label use despite these restrictions, the EMA recommended full suspension of HES marketing authorization across the EU in 2022. Consequently, clinical attention shifted toward alternative colloids, including gelatin-based plasma expanders [11,12].

Gelatin solutions, such as 4% succinylated gelatin, induce transient intravascular volume expansion and have historically been considered safer than HES due to their shorter intravascular half-life and organ-independent metabolism via ubiquitous peptidases [12]. However, robust clinical evidence regarding their safety and efficacy remains limited [13,14]. While gelatin remains in clinical use, several expert statements recommend careful risk–benefit evaluation before use, particularly in critically ill patients without clear hemodynamic indications [15].

Emerging data have raised concerns regarding potential renal effects of synthetic colloids. A large trauma registry analysis (~48,500 patients) reported higher rates of renal failure and dialysis in patients receiving synthetic colloids, particularly when volumes exceeded 1 L [6]. Subsequent ICU trials examining gelatin as a replacement for HES found no renal benefit, and in some cases reported worse renal function and higher mortality compared to crystalloids [16]. Experimental and clinical studies further suggested that gelatin administration may increase urinary biomarkers of tubular stress compared with crystalloid resuscitation [17]. Although the clinical significance of these findings remains uncertain, they reinforce the notion that gelatin colloids can induce or exacerbate renal injury [17].

Additional concerns relate to coagulation and allergenic potential. Gelatin-based solutions can impair clot strength and platelet function, primarily via dilutional mechanisms, and may contribute to coagulopathy in the setting of massive transfusion or volume overload [18]. Anaphylactic reactions appear to occur primarily with urea-linked gelatin preparations, while succinylated gelatin, as used in this study, is associated with a lower risk. However, precise incidence rates for anaphylaxis with succinylated formulations remain unknown.

Nonetheless, gelatin colloids remain widely licensed and used in trauma resuscitation protocols, particularly where rapid volume expansion is required. Given the paucity of trauma-specific data, it is critical to establish whether gelatin can be safely utilized without exacerbating kidney injury or other complications in bleeding trauma patients.

In trauma-related hemorrhagic shock, acute kidney injury is often a consequence of hypovolemia-induced renal hypoperfusion rather than direct nephrotoxicity. From this perspective, gelatin administration may theoretically protect renal function by improving intravascular volume and restoring renal perfusion. This concept is supported by early trauma studies in which colloid-based resuscitation, including hydroxyethyl starch, was associated with less shock-related kidney injury, although these findings must be interpreted in light of later safety concerns regarding synthetic colloids [19].

This retrospective study therefore aimed to evaluate the effect of gelatin administration on the development of AKI among critically ill trauma patients during their first seven days on the ICU. Secondary analyses explored hemodynamic, metabolic and coagulation parameters to determine whether gelatin exposure contributes to renal injury or primarily reflects underlying illness severity.

## 2. Materials and Methods

### 2.1. Study Design and Setting

We conducted a retrospective single-center cohort study evaluating the clinical effects and safety profile of balanced 4% succinylated gelatin (Gelofusine, B. Braun Melsungen AG, Melsungen, Germany) in trauma resuscitation. The study was performed at a level I trauma center (University Hospital Innsbruck, Innsbruck, Austria) and included all eligible trauma patients admitted to the ICU between 1 January 2015 and 1 March 2020.

The study adhered to the RECORD (Reporting of studies Conducted using Observational Routinely collected health Data) recommendations, and the protocol was approved by the local ethics committee of the Medical University of Innsbruck (EK Nr. 1355/2023). All data were extracted from routine electronic hospital records in accordance with local regulations and the Declaration of Helsinki.

### 2.2. Patient Population

All adult trauma patients (≥18 years) admitted to the ICU during the study period were screened for eligibility. The only exclusion criterion was an ICU length of stay shorter than 24 h. No patients were excluded based on injury type or comorbidity. Patient identification was performed using an automated database query based on the predefined eligibility criteria. All identified eligible patients were included in the study cohort, with no additional exclusions after eligibility assessment.

### 2.3. Data Collection

Clinical and laboratory data were extracted from electronic medical records. Recorded variables included demographic characteristics, comorbidities, injury severity, hemodynamic parameters, laboratory measurements, and intravenous fluid administration.

Volumes of gelatin and crystalloid solutions were assessed cumulatively across the pre-ICU phase (including treatment at referring hospitals in transferred patients), emergency department (ER), operating room (OR), and ICU stay. Renal outcomes included serum creatinine, urine output, and Kidney Disease: Improving Global Outcomes (KDIGO)-defined AKI stages. Additional outcome measures included hemodynamic variables, vasopressor requirements, metabolic parameters, and coagulation profiles derived from rotational thromboelastometry (ROTEM).

### 2.4. Statistical Analysis

Continuous variables are presented as median and interquartile range (IQR), whereas categorical variables are reported as counts and percentages. Continuous variables were compared using Mann–Whitney U tests and categorical variables using Fisher’s exact test. Percentages refer to patients with available data for the respective variable.

To explore associations between gelatin exposure and clinical outcomes, mixed-effects regression models with random intercepts for patient and ICU day were used. KDIGO stage was analyzed as both a continuous outcome and as a binary variable (KDIGO ≥ 2 vs. <2). Adjusted models included serum creatinine, lactate, crystalloid volume (mL/kg/day), daily fluid balance, fluid overload > 10% of body weight and AKI at ICU admission (Model B), with additional adjustments for chronic kidney disease (Model C) or sepsis (Model D). Because daily KDIGO ≥ 2 status hardly varied within patients, Model E used patient-level logistic regression for KDIGO stage ≥ 2 on any ICU day, with the mean daily gelatin dose and patient-level covariates. Gelatin effects are reported per 1 mL/kg/day.

Covariates were selected a priori based on clinical knowledge of the determinants of AKI after trauma and of the availability of daily measurements. Repeated measurements were accounted for by random intercepts for patient and ICU day; time-varying covariates (creatinine, lactate, crystalloid volume, daily fluid balance and fluid overload) were entered with their same-day values, and the gelatin dose of the previous day was used in a sensitivity analysis. Fluid overload was calculated as the cumulative ICU fluid balance from ICU day 1 to the respective day (in liters) divided by body weight at admission (in kg) × 100 and dichotomized at >10%; days without documented fluid balance were counted as zero. Collinearity was assessed with variance inflation factors (all ≤ 1.10). KDIGO stage ≥ 2 was chosen as primary endpoint because stage 1 is very common after major trauma and frequently reflects transient, hemodynamically mediated changes in creatinine or urine output; any AKI (KDIGO ≥ 1) and KDIGO stage 3 were analyzed as alternative endpoints. The number of patients and observations contributing to each analysis is given in Supplementary Table S1 and the patient flow in Supplementary Figure S1.

Exploratory clustering used partitioning around medoids (PAM) on a weighted Gower distance. For each patient, the mean and slope over ICU days 1–7 (and, for KDIGO and AKIN, additionally the variance) of KDIGO and AKIN stage, lactate, systolic and diastolic blood pressure, pulse pressure, creatinine, urea, daily fluid balance, CRP, sodium, chloride, fibrinogen and antithrombin were derived, together with chronic kidney disease, AKI at ICU admission and fluid overload > 10%; renal features (KDIGO, creatinine) were weighted most strongly. The number of clusters was chosen based on the mean silhouette width (k = 3:0.47) and clinical interpretability. Gelatin exposure was not used as a clustering feature. Daily associations between gelatin dose and clinical parameters were described with Pearson correlation coefficients; Spearman rank correlations with *p* values adjusted for multiple testing (Benjamini–Hochberg) were calculated as a sensitivity analysis. Statistical significance was defined as a two-tailed *p* value < 0.05.

Missing values were not imputed in the main analyses. Descriptive statistics and group comparisons are based on all patients with available data for the respective variable. The mixed-effects models use all available patient-day observations and provide unbiased estimates under the missing-at-random assumption. Observations after initiation of renal replacement therapy (RRT) were censored, because KDIGO staging and serum creatinine are no longer interpretable under RRT. Reasons for missing daily observations (death, ICU discharge, RRT) are summarized in Supplementary Table S2.

Two sensitivity analyses addressed missing data. First, missing covariate values in the adjusted models were imputed multiple times by chained equations (30 imputations, predictive mean matching) and estimates were pooled using Rubin’s rules. Second, because missing daily KDIGO values resulted mainly from death or ICU discharge, these events were treated as competing events rather than as missing data: time to new-onset AKI (first KDIGO stage ≥ 2 or initiation of RRT on ICU days 2–7) was analyzed with cause-specific Cox models including the gelatin dose of the previous day as a time-dependent covariate, adjusted for SAPS 3 and chronic kidney disease. Cumulative incidence was estimated with the Aalen–Johansen estimator and compared with Gray’s test, and a Fine–Gray model was fitted using the gelatin dose on ICU day 1. Patients with AKI on ICU day 1 were excluded from this analysis.

Further sensitivity analyses comprised (i) exclusion of patients with AKI at ICU admission, (ii) cumulative gelatin exposure (cumulative dose up to the previous day as time-dependent covariate, cumulative dose to date in the linear mixed model, and tertiles of the day-1 dose), (iii) effect modification by age, illness severity, shock severity (emergency-room lactate ≥ 2 mmol/L, emergency-room systolic blood pressure < 90 mmHg), creatinine at ICU admission, chronic kidney disease and fluid overload, tested by interaction terms in Model B, (iv) RRT initiation on ICU days 2–7 as secondary outcome (cause-specific Cox model with the gelatin dose of the previous day), and (v) an inverse-probability-weighted analysis of high gelatin exposure on ICU day 1 (≥10 mL/kg). Stabilized weights were derived from a propensity score including age, sex, SAPS 3, ISS, chronic kidney disease, AKI at ICU admission, creatinine at ICU admission, emergency-room lactate and systolic blood pressure, base excess at ICU admission and polytrauma, and were truncated at the 1st and 99th percentiles. A comparison with patients who never received gelatin was not possible, because almost all patients were exposed.

Analyses were conducted using R version 4.2.1 (sensitivity analyses: R 4.3.3 with the packages lme4, mice, survival and cmprsk) and GraphPad Prism 9 version 9.0.2 (GraphPad Software, La Jolla, CA, USA). Cluster visualizations and time-series analyses were generated using ggplot2 and related tidyverse packages for R version 4.2.1.

## 3. Results

### 3.1. Baseline Patient Characteristics and AKI Incidence

Of 220 trauma patients, 5 had no AKI staging available during ICU days 1–7 (in three patients RRT was started on the day of ICU admission); the analysis cohort therefore comprised 215 critically ill trauma patients (Table 1). Median age was 51 years (IQR 34.5–66.5), and 25% were female. Blunt trauma accounted for the majority of injuries (96%), and 74.4% of patients sustained polytrauma, defined as multiple injury with an ISS ≥ 16. The median ISS was 24 (IQR 16–34), and median Simplified Acute Physiology Score 3 (SAPS 3) on ICU admission was 48 (IQR 36–60.5).

During the first week of ICU treatment, 36 patients (17%) had KDIGO stage ≥ 2 AKI. Patients who developed AKI were significantly older (median 65.5 vs. 49 years, *p* < 0.001), had higher SAPS 3 scores (59 vs. 46, *p* < 0.001), and presented more frequently with cardiovascular (66.7% vs. 21.2%, *p* < 0.001), coagulation (41.7% vs. 13.4%, *p* < 0.001), renal (27.8% vs. 6.7%, *p* < 0.001), hepatic (19.4% vs. 5.0%, *p* = 0.008), and central nervous system (CNS) comorbidities (27.8% vs. 12.3%, *p* = 0.036). Polytrauma was less frequent among patients who developed AKI (55.6% vs. 78.2%, *p* = 0.007).

AKI was already present at ICU admission in 27 patients (12.6%). Of the 36 patients with KDIGO stage ≥ 2, 15 met this criterion on ICU day 1 and 21 developed it on days 2–7 (17 de novo and 4 by progression of AKI present at admission). Within the first seven ICU days, 15 patients (7.0%) required RRT, and urine output below 0.5 mL/kg/h for at least 6 h was documented in 36 of 177 patients with available data (20.3%). ICU mortality was 27.8% in patients with and 10.1% in patients without AKI (*p* = 0.011) (Supplementary Table S3).

AKI patients exhibited higher initial serum creatinine levels on ICU admission (1.16 vs. 0.96 mg/dL, *p* < 0.001) and reduced estimated glomerular filtration rate (GFR) at all time points (ICU: 58 vs. 60 mL/min/1.73m^2^, *p* < 0.001). Lactate in the emergency room was markedly elevated in AKI patients (45 vs. 20 mg/dL, corresponding to 5.0 vs. 2.2 mmol/L, *p* = 0.001), and base excess was more negative (−3.6 vs. −1.3 mmol/L, *p* < 0.001). Systolic and diastolic blood pressures at the prehospital scene were significantly lower among AKI patients (e.g., systolic: 90 vs. 120 mmHg, *p* < 0.001), reflecting more severe hemodynamic compromise.

No significant differences were observed in gelatin administered at referring hospitals or in the emergency room administration between patients with and without AKI. Intraoperative gelatin volumes were significantly lower in AKI patients (500 vs. 1000 mL, *p* = 0.007). Volumes of the balanced crystalloid ELO-MEL administered at referring hospitals did not differ significantly (500 vs. 0 mL, *p* = 0.104), whereas intraoperative volumes were lower in AKI patients (1000 vs. 1505 mL, *p* = 0.026); total crystalloid volumes showed the same pattern (intraoperative 1002 vs. 1544 mL, *p* = 0.051). These findings suggest that fluid exposure patterns differed early between groups, potentially reflecting clinical decision-making in response to illness severity rather than causative effects.

Gelatin was administered to 209 of the 215 patients (97.2%) before or during the first ICU week (159 before ICU admission, 194 on ICU day 1); only six patients never received gelatin. Among recipients, the median cumulative ICU dose over days 1–7 was 51.5 mL/kg (IQR 25.6–80.4).

### 3.2. Relationship Between Gelatin Administration and Kidney Injury

A series of mixed-effects regression models was used to examine the relationship between gelatin dose (mL/kg/day) and KDIGO over time (Table 2).

In the unadjusted model (model A), gelatin administration was not associated with KDIGO progression. After adjustment for serum creatinine, lactate, crystalloid volume, daily fluid balance, fluid overload > 10% and AKI at ICU admission (model B), gelatin exposure remained not significantly associated with KDIGO stage. The lack of a statistically significant association persisted in models additionally adjusted for chronic kidney disease (Model C) and sepsis (Model D). In Model E, clinically relevant kidney injury was assessed by using KDIGO stage ≥ 2 as the outcome, showing no significant association between gelatin and development of moderate-to-severe AKI (OR 1.039 per 1 mL/kg/day; 95% CI 0.995 to 1.086).

In the adjusted models, higher KDIGO stages were associated with higher serum creatinine (*p* < 0.001), AKI at ICU admission (*p* < 0.001) and higher crystalloid volumes (*p* = 0.006), with a trend for fluid overload > 10% (*p* = 0.068); lactate and daily fluid balance were not independently associated. Gelatin exposure did not emerge as an independent predictor of KDIGO progression.

Sensitivity analyses confirmed these findings. Excluding creatinine from the adjustment set (to avoid over-adjustment for a component of the KDIGO definition) and using the gelatin dose of the previous day yielded similar results (β = −0.0010, 95% CI −0.003 to 0.0009, and β = −0.0006, 95% CI −0.0023 to 0.0011, per 1 mL/kg/day). After multiple imputation of missing covariates, gelatin remained unassociated with KDIGO stage (Model B: β = −0.0006; 95% CI −0.0024 to 0.0011; *p* = 0.474). In the competing-risk analysis (196 patients without AKI on ICU day 1), 23 patients developed new-onset AKI, 12 died and 67 were discharged from the ICU by day 7. The gelatin dose of the previous day was not associated with the hazard of new-onset AKI (adjusted cause-specific HR 0.997 per 1 mL/kg; 95% CI 0.976 to 1.019; *p* = 0.779), and the day-1 gelatin dose was not associated with the cumulative incidence of AKI (sub-distribution HR 1.001; 95% CI 0.985 to 1.018; *p* = 0.870). Higher gelatin doses were, however, associated with a lower rate of ICU discharge (adjusted cause-specific HR 0.919; 95% CI 0.887 to 0.952) (Supplementary Tables S4 and S5a–c, Supplementary Figure S2).

Regarding the magnitude of effects compatible with our data, the upper 95% confidence limit of Model B corresponds to an increase of at most 0.016 KDIGO stages per additional 10 mL/kg/day of gelatin, and that of the competing-risk analysis to a hazard ratio of 1.21 per additional 10 mL/kg. The patient-level logistic model was less precise (OR 1.039 per mL/kg/day; 95% CI 0.995–1.086, i.e., up to 2.3 per additional 10 mL/kg/day), so that a clinically relevant harmful effect cannot be excluded. Results were similar after exclusion of the 27 patients with AKI at ICU admission (Model B: β = −0.0011; 95% CI −0.0029 to 0.0007; Model E: OR 1.051; 95% CI 0.971–1.138) and with any AKI as endpoint (KDIGO ≥ 1, 70 of 199 patients: OR 1.034; 95% CI 0.992–1.077). For KDIGO stage 3 (21 events, adjusted for creatinine and AKI at admission only) the estimate was of similar size and reached nominal significance (OR 1.037; 95% CI 1.001–1.073; *p* = 0.042).

Cumulative gelatin exposure was not associated with new-onset AKI (adjusted HR 0.997 per mL/kg; 95% CI 0.980–1.014), and the incidence of new-onset AKI did not increase across tertiles of the day-1 dose (6.2%, 15.4% and 12.9%; *p* for trend = 0.465). In the linear mixed model, however, the cumulative dose to date was associated with higher KDIGO stages (β = 0.0021 per mL/kg; 95% CI 0.0007–0.0034; *p* = 0.003), which may reflect the longer duration of hemodynamic instability in patients receiving repeated doses. RRT was started in 10 patients on ICU days 2–7; the gelatin dose of the previous day was not associated with RRT initiation (HR 1.009 per mL/kg; 95% CI 0.978–1.041; adjusted for SAPS 3: 0.978; 95% CI 0.949–1.007), although this analysis is limited by the small number of events. No effect modification was found for age, illness severity, shock severity, chronic kidney disease or fluid overload (all *p* for interaction ≥ 0.08); in patients with creatinine > 1.2 mg/dL at ICU admission, the gelatin estimate was negative (*p* for interaction < 0.001). In the inverse-probability-weighted analysis (172 of 208 patients with ≥ 10 mL/kg on ICU day 1; standardized mean differences after weighting ≤ 0.21), the odds ratios for KDIGO stage ≥ 2 (1.75; 95% CI 0.31–9.71) and for new-onset AKI (1.28; 95% CI 0.16–10.13) were very imprecise (Supplementary Table S6).

### 3.3. Renal Phenotyping by Unsupervised Cluster Analysis

To explore distinct renal trajectories, exploratory unsupervised clustering using longitudinal physiological data from the first ICU week was performed. Variables included KDIGO and AKIN scores, blood pressure (systolic, diastolic and pulse pressure), lactate, fluid balance, fluid overload > 10%, serum creatinine, urea, CRP, electrolytes (Na^+^, Cl^−^), fibrinogen and antithrombin, as well as chronic kidney disease and AKI at ICU admission. Variables directly related to renal function were given higher weighting in the clustering algorithm. For each parameter, the mean and trend (slope) over time, and for KDIGO and AKIN additionally the variance, were extracted to characterize patient-level patterns. Importantly, gelatin exposure was not included in the clustering features to avoid circular interpretation. Three robust phenotypes emerged (Figure 1) (partitioning around medoids; mean silhouette width 0.47).

Cluster 1 (*n* = 5) was characterized by persistently elevated KDIGO scores, representing early and sustained severe AKI. Cluster 2 (*n* = 142) showed stable, low KDIGO stages throughout the ICU stay and reflected preserved kidney function. Cluster 3 (*n* = 26) exhibited progressively increasing KDIGO scores over time, indicating delayed-onset or evolving AKI.

The cluster analysis included 173 patients. Patients who could not be included (*n* = 42) had no repeated measurements after ICU day 1 (median ICU stay 1 day); compared with included patients they had a lower ISS and higher rates of AKI at ICU admission (28.2% vs. 9.1%) and ICU mortality (30.8% vs. 9.1%) (Supplementary Table S7). In bootstrap resampling, Cluster 2 was highly stable (mean Jaccard coefficient 0.92), Cluster 3 moderately stable (0.65) and Cluster 1 unstable (0.36); findings regarding Cluster 1 are therefore exploratory.

Importantly, gelatin exposure was not included in the clustering algorithm to avoid circularity. Post hoc comparison, however, showed that patients in Clusters 1 and 3—those with more severe or progressive AKI—had received higher gelatin volumes (mean daily dose, median 12.8 mL/kg/day in Cluster 3 and 10.0 mL/kg/day in Cluster 1 vs. 8.8 mL/kg/day in Cluster 2; Kruskal–Wallis *p* = 0.006). This raised the question of whether gelatin exposure contributed to worsening AKI or merely reflected underlying severity.

To better understand this relationship, we analyzed supporting hemodynamic, metabolic, and fluid-related variables across clusters (Figure 2).

Across the first ICU week, the three data-derived clusters demonstrated clearly distinct clinical trajectories. Cluster 1 (red) was characterized by the most negative base excess values early after ICU admission, indicating pronounced metabolic acidosis. Lactate levels were moderately elevated but showed signs of recovery over time. Cluster 2 (green) represented the most clinically stable phenotype, with the lowest crystalloid and gelatin requirements, nearly neutral fluid balance, stable blood pressures, and minimal metabolic or inflammatory disturbance. In contrast, Cluster 3 (blue) exhibited the highest cumulative gelatin exposure and the most sustained fluid overload, with persistently positive fluid balance, elevated lactate levels, suppressed antithrombin activity, and elevated CRP values, consistent with ongoing circulatory and inflammatory stress. These patterns suggest that Clusters 1 and 3 reflect distinct forms of hemodynamic and metabolic compromise, while gelatin exposure is more likely to mirror the severity of illness than act as an independent driver of kidney injury.

### 3.4. Correlation Analysis Between Gelatin Administration and Clinical Parameters

To explore the physiological context in which gelatin was administered, daily correlations between its dosage and a range of clinical variables over the first seven ICU days were analyzed (Table 3).

Gelatin dose showed strong positive correlations with cumulative fluid balance and crystalloid administration, particularly during the early resuscitation phase, most pronounced on ICU days 1 (r = 0.41, *p* < 0.001) and 2 (r = 0.52, *p* < 0.001). Gelatin volume was also positively associated with crystalloid volume across all days, peaking on day 4 (r = 0.32, *p* < 0.001).

Gelatin was also associated with markers of circulatory instability: Systolic blood pressure demonstrated negative correlations with gelatin administration from day 1 onward (e.g., r = −0.29 on day 6, *p* < 0.01), while lactate levels were positively correlated on days 2, 5 and 6 (r = 0.18–0.30, *p* < 0.05). Coagulation markers however showed inverse associations, with antithrombin and fibrinogen showing significant negative correlations (e.g., day 2: r = −0.46, *p* < 0.001; r = −0.26, *p* < 0.001).

In contrast, correlations between gelatin exposure and creatinine as well as KDIGO stages were weak or absent, apart from a modest positive association with KDIGO observed only on day 7 (r = 0.21, *p* < 0.05), which did not remain significant after correction for multiple testing, while no consistent pattern emerged across earlier days. No clear relationship between gelatin dose and creatinine or KDIGO stage was observed during the ICU course.

### 3.5. Additional Safety Signals Associated with Gelatin

Beyond renal outcomes, potential safety concerns of gelatin administration—particularly coagulation and hypersensitivity reactions—were evaluated.

Coagulation impairment was evaluated using FIBTEM maximum clot firmness (MCF) measurements over the first ICU week via a reduced mixed-effect model due to unavailability of daily ROTEM analyses. In this model, gelatin dosage did not demonstrate a significant effect on clot firmness (−0.029 mm per mL/kg/day; 95% CI −0.067 to 0.008; *p* = 0.124; Table 4). In contrast, higher albumin and crystalloid administration were both associated with reductions in FIBTEM MCF, suggesting a dilutional or physiologic influence of these fluids on coagulation.

Four anaphylactoid reactions occurred during the study period. Confirmed triggers included erythrocyte transfusions and cefuroxime administration. One intraoperative reaction occurred in the presence of both gelatin and cefuroxime and could not be definitively attributed to either agent. No confirmed gelatin-induced anaphylaxis was identified.

## 4. Discussion

In this retrospective cohort of critically ill trauma patients, administration of 4% succinylated gelatin—at doses frequently exceeding 10 mL/kg/day—was not associated with an increased risk of moderate-to-severe AKI during the first week of intensive care unit (ICU) treatment. Mixed-effects regression models, competing-risk analyses, correlation analyses, and exploratory unsupervised clustering all indicated that gelatin exposure did not independently predict AKI development or progression of KDIGO staging.

These findings are important given the longstanding uncertainty regarding the renal safety profile of gelatin-based colloids. While some observational studies and biomarker analyses have raised concerns about potential nephrotoxic effects—particularly at higher doses or in postoperative settings [12,17]—the clinical relevance of these signals has remained unclear. In contrast, other recent cohort studies have reported comparable AKI and renal replacement therapy (RRT) rates between gelatin and crystalloid-treated patients, while also observing lower cumulative fluid balances in the gelatin groups [20]. This suggests that gelatin may support more efficient volume expansion without increasing renal risk. Consistent with this hypothesis, the GENIUS trial in septic patients found that gelatin use enabled faster hemodynamic stabilization with lower cumulative fluid volumes compared to crystalloids, although without significant differences in renal outcomes [7].

Only in this broader context does the experience with hydroxyethyl starch (HES) offer contrast. HES was withdrawn from most clinical settings after randomized trials demonstrated increased 90-day mortality and higher RRT rates, particularly in sepsis [21,22]. Unlike HES, modern gelatin solutions are rapidly cleared, have lower molecular weight, and lack osmotic nephrosis potential—factors that may contribute to their more favorable renal safety profile [7,14].

Our results provide additional clinical evidence in a trauma population. Even at doses exceeding 10 or even 30 mL/kg/day, gelatin administration was not directly associated with increased rates of moderate-to-severe AKI among critically ill severe trauma patients, with multiple mixed-effect regression models showing no significant association between gelatin use and AKI progression. Moreover, our data suggest that the development of AKI among this cohort was primarily associated with early hemodynamic impairment and development of volume overload. These findings are consistent with emerging observational data suggesting that modern gelatin solutions, unlike HES, do not confer a substantial nephrotoxic burden in vivo when used judiciously [7,20].

Furthermore, our multivariable analysis highlights the importance of patient-specific risk factors [20,21]. These findings suggest that gelatin’s renal impact, if any, is largely overshadowed by the patient’s intrinsic renal reserve and the severity of the initial insult. While it did not inherently worsen renal outcomes on a population level, our results support the continued practice of tailoring fluid therapy to patient risk. Particularly among patients on the brink of kidney injury or with pre-existing kidney impairment, even transient decreases in renal function may precipitate or worsen an episode of AKI.

To further explore phenotypic differences in renal trajectories, we performed unsupervised clustering using longitudinal physiologic data. This analysis identified three distinct patterns: Cluster 1 featured early and persistent severe AKI with elevated fluid requirements during the first ICU days. Cluster 2, representing the most stable patients, showed preserved kidney function with minimal fluid accumulation and lowest gelatin exposure. Cluster 3 displayed delayed or progressive AKI development alongside sustained positive fluid balance and elevated lactate levels. Although gelatin exposure was not included in the clustering algorithm, post hoc analysis revealed higher gelatin doses in the AKI-prone clusters. These findings underscore that gelatin administration likely reflects clinical response to underlying hemodynamic instability rather than constituting an independent renal risk factor.

While our analysis showed consistent positive correlations between gelatin dose and markers of hemodynamic instability, as well as fluid balance, no clear relationship between gelatin dose and creatinine or KDIGO stage could be observed over the ICU course. This suggests that while gelatin may be used more frequently in sicker patients, it is not an independent driver of renal injury. This interpretation is supported by the competing-risk analysis: patients receiving higher gelatin doses were discharged from the ICU considerably later, whereas the gelatin dose was not associated with the rate of new-onset AKI after adjustment for illness severity. While gelatin can be part of the armamentarium for trauma resuscitation, careful attention to volume status and early signs of renal stress is warranted, especially in those patients with compromised renal function.

As hemostasis poses an essential goal in trauma management, coagulopathy among this cohort was also addressed, showing a dose-dependent reduction in fibrin-based clot strength under increasing volumes of resuscitation fluids alongside significantly lower levels of fibrinogen and antithrombin. Although gelatin had no significant effect on clot firmness in our model, dilutional coagulopathy is well described with colloid infusions, resulting from hemodilution of clotting factors and possibly gelatin-induced platelet dysfunction as shown in vitro and in vivo [23,24].

While we found no evidence of nephrotoxicity, it is not entirely benign: its use requires balancing the benefits of volume expansion against potential impacts on coagulation. Although shock and hypovolemia are counted among the most important reasons for trauma induced coagulopathy, this side effect deserves consideration. Using lowest effective doses of gelatin alongside supplementation with blood products or fibrinogen concentrate may mitigate these issues. Current European trauma guidelines already emphasize judicious use of fluids to avoid exacerbating coagulopathy, and our data reinforce that message in the context of gelatin.

This study has several limitations: First retrospective, single-center design does not allow causal inference, and gelatin was administered at the discretion of the treating physicians; despite multivariable adjustment, residual confounding by indication is likely and there were no pre-trauma measurements available reflecting baseline renal function. Furthermore, the limited temporal resolution of the available data did not allow reliable differentiation between variables measured before and after initial gelatin administration, potentially introducing bias through adjustment for post-exposure variables. Additionally, the outcomes of our analyses were focused only on AKI events with KDIGO stage ≥ 2 occurring within the first seven ICU days, which were limited (36 patients, of whom 23 developed new-onset AKI after ICU day 1), resulting in wide confidence intervals. Hence the logistic model cannot exclude a clinically relevant risk and no statement about later AKI or long-term renal dysfunction can be made. The absence of a statistically significant association should therefore not be interpreted as proof of renal safety. Daily observations were frequently missing after ICU discharge or death; as these events are likely related to illness severity, missingness may not be completely at random, although a competing-risk analysis yielded consistent results (Supplementary Table S5a). Cluster analysis was exploratory and one cluster comprised only five patients, allowing only limited explanatory power. Furthermore, the limited sample size precluded a reliable assessment of rare adverse events such as anaphylaxis, while coagulation-related effects were evaluated using FIBTEM MCF rather than clinical bleeding outcomes. Lastly, gelatin volumes administered before ICU admission were incompletely documented, particularly for patients transferred from referring hospitals. Because almost all patients received gelatin, a comparison with unexposed patients was not possible, and the propensity-score-weighted comparison of high versus lower exposure yielded very imprecise estimates. Finally, the correlation and cluster analyses were exploratory, and one of the three clusters was unstable in bootstrap resampling.

## 5. Conclusions

In this retrospective cohort of severely injured trauma patients, administration of 4% succinylated gelatin was not associated with an increased risk of moderate-to-severe AKI during the first week of ICU care. Observed renal function changes were more consistent with the physiological consequences of hemodynamic instability and fluid balance than with intrinsic nephrotoxicity of gelatin.

These findings provide relevant clinical evidence to the ongoing debate about colloid safety. Nonetheless, the absence of a nephrotoxic signal in our study should not engender complacency: close monitoring of renal function and volume status remains imperative, especially in patients with pre-existing renal impairment. Future prospective research in trauma patients will be necessary to verify these findings and to further define the renal safety profile of gelatin. Future adequately powered prospective multicenter studies should particularly address subgroups at greater risk for renal impairment, using standardized exposure definitions and prespecified renal outcomes, including longer-term kidney function. Until then, our findings should be interpreted cautiously and should not be considered evidence of renal safety.

## Figures and Tables

**Figure 1:**
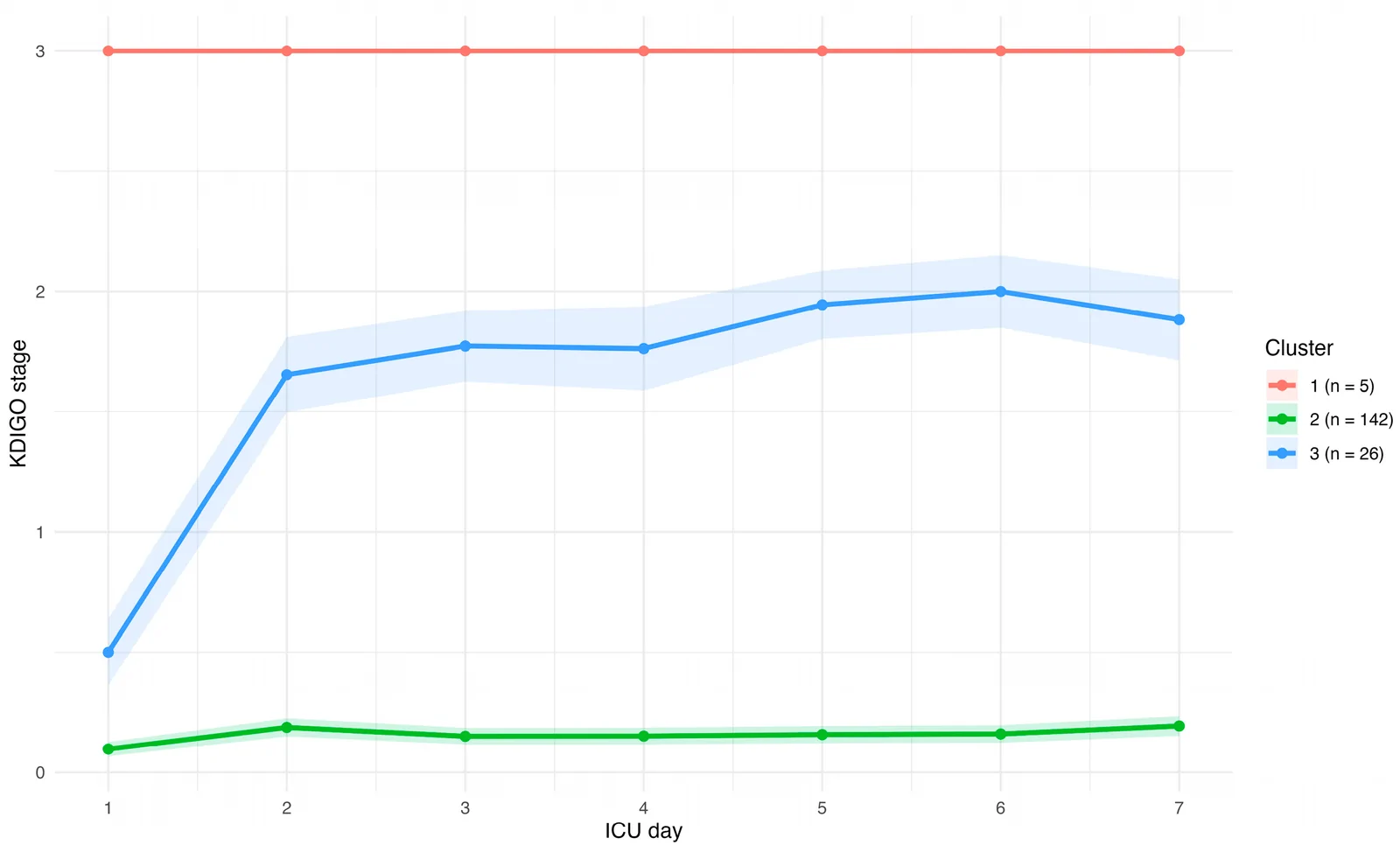
Trajectories of KDIGO stages across the first ICU week stratified by patient clusters. Clustering based on ICU day 1–7 trajectories of renal and hemodynamic parameters (i.e., KDIGO, AKIN, lactate, blood pressure, creatinine, fluid status, electrolytes, inflammation markers). Gelatin exposure was intentionally excluded from feature construction to allow unbiased post hoc comparison between clusters.

**Figure 2:**
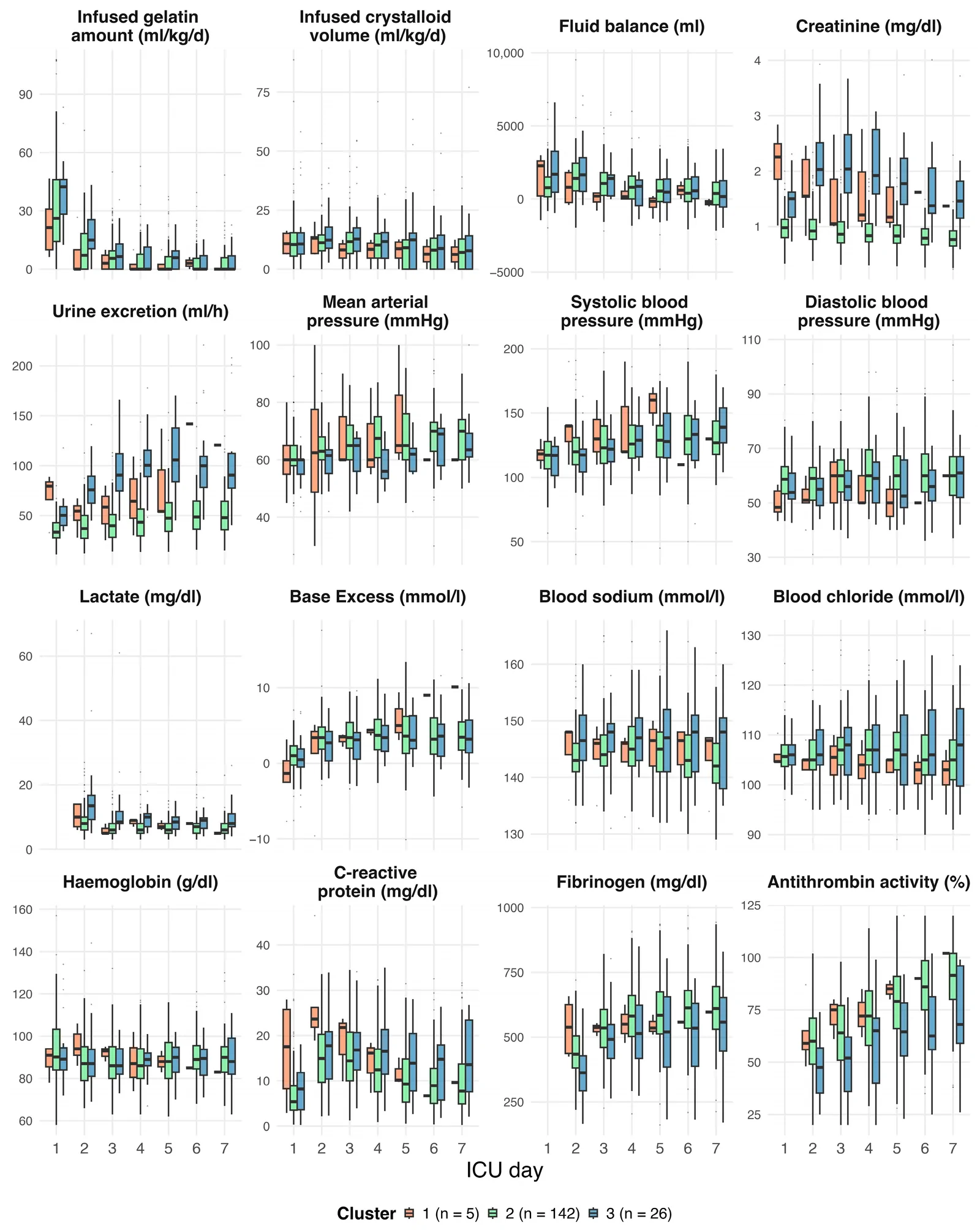
Temporal patterns of ICU variables by feature-based patient clustering. Clustering trajectories of hemodynamic, laboratory and renal functional parameters over the course of the ICU days 1 to 7, stratified by patient clusters (1 = red, 2 = green, 3 = blue). Corresponding parameters and units of *y*-axis scaling stated in parentheses above each respective graph.

**Table 1:** Baseline patient characteristics stratified by the occurrence of acute kidney injury (AKI) during the first ICU week. Continuous variables are reported as median and interquartile range (IQR), categorical variables as number and percentage. AKI was defined as KDIGO stage ≥ 2. *p* values were calculated using the Mann–Whitney U test for continuous variables and Fisher’s exact test for categorical variables. Percentages refer to patients with available data. Five of 220 trauma patients without AKI staging during ICU days 1–7 are not included.

	Total (Median/IQR or *n*/N/%) *n* = 215	No AKI (Median/IQR or *n*/%) *n* = 179	AKI (Median/IQR or *n*/%) *n* = 36	*p* Value	Missing, *n* (%)
**Age**	51 (34.5–66.5)	49 (31.5–60)	65.5 (54–79)	<0.001	0 (0.0%)
**Weight (kg)**	77 (70–90)	77 (70–90)	77.5 (70–87.25)	0.958	6 (2.8%)
**Height (cm)**	175 (170–180)	175 (170–180)	174 (166.25–180)	0.087	6 (2.8%)
**BMI**	24.69 (23.15–27.7)	24.69 (22.94–27.22)	25.71 (23.72–28.88)	0.323	6 (2.8%)
**Sex (female)**	53/215 (24.7%)	41/179 (22.9%)	12/36 (33.3%)	0.206	0 (0.0%)
**Co-morbidity CNS**	32/215 (14.9%)	22/179 (12.3%)	10/36 (27.8%)	0.036	0 (0.0%)
**Co-morbidity cardiovascular**	62/215 (28.8%)	38/179 (21.2%)	24/36 (66.7%)	<0.001	0 (0.0%)
**Co-morbidity liver**	16/215 (7.4%)	9/179 (5%)	7/36 (19.4%)	0.008	0 (0.0%)
**Co-morbidity kidney**	22/215 (10.2%)	12/179 (6.7%)	10/36 (27.8%)	<0.001	0 (0.0%)
**Co-morbidity coagulation**	39/215 (18.1%)	24/179 (13.4%)	15/36 (41.7%)	<0.001	0 (0.0%)
**Co-morbidity active oncologic disease**	4/215 (1.9%)	1/179 (0.6%)	3/36 (8.3%)	0.015	0 (0.0%)
**Co-morbidity other**	122/215 (56.7%)	92/179 (51.4%)	30/36 (83.3%)	<0.001	0 (0.0%)
**Trauma**	215/215 (100%)	179/179 (100%)	36/36 (100%)	NA, only one level	0 (0%)
**Polytrauma**	160/215 (74.4%)	140/179 (78.2%)	20/36 (55.6%)	0.007	0 (0.0%)
**Burn trauma**	9/215 (4.2%)	7/179 (3.9%)	2/36 (5.6%)	0.648	0 (0.0%)
**Blunt injury**	190/197 (96.4%)	161/167 (96.4%)	29/30 (96.7%)	1.000	18 (8.4%)
**Penetrating injury**	3/196 (1.5%)	3/166 (1.8%)	0/30 (0%)	1.000	19 (8.8%)
**Systolic blood pressure (emergency doctor)**	120 (99.75–140)	120 (100–140)	90 (80–120)	<0.001	59 (27.4%)
**Diastolic blood pressure (emergency doctor)**	70 (60–80)	70 (60–80)	60 (40–68.5)	<0.001	74 (34.4%)
**Systolic BP (ER)**	117 (100–140)	120 (100–140)	105 (90–120)	0.056	29 (13.5%)
**Systolic BP (prior to ICU)**	120 (110–120)	120 (105–125)	115 (110–120)	0.922	204 (94.9%)
**Systolic BP (ICU)**	120 (100–136)	120 (102–136.5)	120 (98–132.75)	0.480	0 (0.0%)
**Diastolic BP (ER)**	64.5 (51.25–80)	65 (55–80)	60 (50–70)	0.200	29 (13.5%)
**Diastolic BP (prior to ICU)**	60 (57.5–60)	60 (60–60)	55 (50–62.5)	0.408	204 (94.9%)
**Diastolic BP (ICU)**	60 (50–72)	62 (50–73)	58 (47.5–68.25)	0.038	0 (0.0%)
**ISS Score DGU**	24 (16–34)	24 (17–34)	22 (9–39.5)	0.554	1 (0.5%)
**TASH Score (emergency room)**	6 (4–11)	6 (3–10)	12 (5–14.5)	0.003	49 (22.8%)
**SAPS 3 Score (ICU admission)**	48 (36–60.5)	46 (34–58)	59 (50–73)	<0.001	20 (9.3%)
**Creatinine (ER)**	1.02 (0.86–1.19)	1 (0.84–1.17)	1.16 (0.92–1.43)	0.043	28 (13.0%)
**Creatinine (prior to ICU)**	1 (0.84–1.56)	0.93 (0.8–1.03)	3.14 (1.4–3.98)	<0.001	186 (86.5%)
**Creatinine (ICU)**	0.97 (0.86–1.16)	0.96 (0.81–1.12)	1.16 (0.94–1.5)	<0.001	62 (28.8%)
**GFR (ER)**	60 (60–60)	60 (60–60)	60 (52–60)	0.017	42 (19.5%)
**GFR (prior to ICU)**	60 (42–60)	60 (60–60)	25 (20–54)	<0.001	190 (88.4%)
**GFR (ICU)**	60 (60–60)	60 (60–60)	58 (46–60)	<0.001	74 (34.4%)
**Base excess (ICU)**	−1.55 (−3.58–0.2)	−1.3 (−3.15–0.35)	−3.6 (−5.7–−1.95)	<0.001	9 (4.2%)
**Lactate, mg/dL (ER)**	21 (12–35)	20 (11–31)	45 (17.5–75.25)	0.001	61 (28.4%)
**Gelatin (referring hospital, T-2)**	0 (0–0)	0 (0–0)	0 (0–0)	0.836	24 (11.2%)
**Gelatin (ER)**	500 (0–1000)	500 (0–1000)	0 (0–500)	0.532	23 (10.7%)
**Gelatin (OR)**	1000 (500–2000)	1000 (500–2000)	500 (75–1000)	0.007	92 (42.8%)
**ELO-MEL (referring hospital, T-2)**	250 (0–500)	0 (0–500)	500 (0–1000)	0.104	24 (11.2%)
**ELO-MEL (ER)**	500 (0–1000)	500 (0–1000)	250 (0–1000)	0.225	24 (11.2%)
**ELO-MEL (OR)**	1500 (900–2502.5)	1505 (1000–2800)	1000 (505–1800)	0.026	91 (42.3%)
**Crystalloids total (referring hospital, T-2)**	500 (0–1000)	500 (0–1000)	500 (375–1000)	0.103	24 (11.2%)
**Crystalloids total (ER)**	500 (500–1000)	500 (500–1000)	500 (0–1000)	0.362	24 (11.2%)
**Crystalloids total (OR)**	1502.5 (980.75–2532.5)	1544.1 (1000–2800)	1001.85 (555–2000)	0.051	91 (42.3%)

**Abbreviations:** AKI = acute kidney injury; BMI = Body Mass Index; CNS = central nervous system; DGU = Deutsche Gesellschaft für Unfallchirurgie; ER = emergency room; GFR = glomerular filtration rate according to CKD-EPI creatinine equation (2009) (values censored above 60 mL/min/1.73 m^2^); ICU = intensive care unit; ISS = Injury Severity Score; SAPS = Simplified Acute Physiology Score; TASH = Trauma-Associated Severe Hemorrhage. Lactate in mg/dL (divide by 9.01 for mmol/L). ER = T-1A (emergency room); OR = T-1B (operating room); prior to ICU = T-1C; ICU = ICU admission (T0); referring hospital = T-2 (>24 h before ICU admission, transferred patients only). ELO-MEL = balanced crystalloid solution; total crystalloids = all crystalloid solutions, restricted to patients with the respective time point documented.

**Table 2:** Association between gelatin exposure and KDIGO stage across different mixed-effects regression models. Model A: unadjusted; Model B: adjusted for serum creatinine, lactate, crystalloid volume, daily fluid balance, fluid overload > 10%, and AKI at ICU admission; Model C: Model B + chronic kidney disease; Model D: Model B + sepsis; Model E: logistic regression for KDIGO stage ≥ 2 on any ICU day (moderate/severe AKI), adjusted as Model B. Estimates reflect the effect per 1 mL/kg/day of gelatin. β for Models A–D (linear mixed-effects models with random intercepts for patient and ICU day); odds ratio for Model E (patient level, per 1 mL/kg/day of mean daily dose).

Model	Gelatin Estimate (β or OR)	95% CI	*p* Value	Patients/Observations
Model A: KDIGO (continuous), unadjusted	−0.0017	(−0.0036 to 0.0001)	0.065	207/1047
Model B: KDIGO (continuous), adjusted	−0.0002	(−0.0020 to 0.0016)	0.840	199/970
Model C: Model B + chronic kidney disease	−0.0001	(−0.0019 to 0.0017)	0.887	199/970
Model D: Model B + sepsis	−0.0002	(−0.0020 to 0.0016)	0.795	199/969
Model E: KDIGO ≥ 2 on any ICU day (logistic, OR)	1.039	(0.995 to 1.086)	0.086	199/–

**Table 3:** Correlation with amount of gelatin (mL/kg/day). Values represent Pearson correlation coefficients for each variable on ICU days 1 through 7. Spearman rank correlations with *p* values adjusted for multiple testing (Benjamini–Hochberg) are provided in Supplementary Table S8. Statistical significance (unadjusted) is indicated by symbols at the correlation coefficients: *p* < 0.05 (*), *p* < 0.01 (**), and *p* < 0.001 (***). Positive correlations indicate higher values of the respective parameters in association with higher gelatin doses, whereas negative correlations reflect inverse relationships. This analysis is descriptive and not adjusted for confounding.

Variable	Day 1	Day 2	Day 3	Day 4	Day 5	Day 6	Day 7
**KDIGO**	−0.027	0.082	0.007	−0.090	0.103	0.165	0.207 *
**AKIN**	−0.046	0.042	0.211	−0.023	0.131	0.151	0.333 ***
**Diastolic BP**	−0.077	−0.218 **	−0.095	−0.100	−0.131	0.028	−0.074
**Systolic BP**	−0.192 *	−0.113	−0.090	−0.170 *	−0.178 *	−0.292 **	−0.152
**Lactate**	NA	0.296 ***	0.115	0.058	0.183 *	0.257 **	0.155
**Creatinine**	0.017	0.060	−0.004	−0.034	0.059	0.118	0.215 *
**Fluid Balance**	0.406 ***	0.518 ***	0.297 ***	0.102	0.195 *	0.190 *	0.082
**Na+**	NA	0.159 *	0.038	0.101	0.068	0.210 *	0.099
**Cl−**	0.317 ***	0.128	0.053	0.057	0.058	0.229 *	0.032
**CRP**	−0.291 **	−0.115	0.037	−0.220 **	−0.036	0.234 *	0.188 *
**Fibrinogen**	NA	−0.263 ***	−0.136	−0.341 ***	−0.243 ***	0.001	−0.032
**Urine Output**	−0.108	0.048	−0.138	−0.078	−0.142	0.060	0.070
**Antithrombin**	NA	−0.462 ***	−0.314 ***	−0.329 ***	−0.379 ***	−0.430 ***	−0.253 **
**Crystalloids**	0.226 **	0.286 ***	0.237 **	0.316 ***	0.199 *	0.153	0.146
**Base Excess**	−0.104	−0.063	−0.204 *	−0.032	−0.130	−0.110	−0.052
**Hemoglobin**	−0.030	0.061	0.026	0.076	−0.135	−0.019	0.048
**Renal SOFA**	0.048	0.096	−0.010	−0.073	0.021	0.084	0.143
**MAP**	−0.189 *	−0.276 **	−0.185	−0.200	−0.266 *	−0.192	−0.213

**Abbreviations:** AKIN = acute kidney injury network; BP = blood pressure; CRP = C-Reactive Protein; KDIGO = Kidney Disease: Improving Global Outcomes; MAP = Mean Arterial Pressure; SOFA = Sequential Organ Failure Assessment.

**Table 4:** Estimated effect of fluids on FIBTEM maximum clot firmness (MCF). Linear mixed-effects model with random intercepts for patient and ICU day. Fluid variables were selected by stepwise reduction; gelatin was retained in the model irrespective of statistical significance. Albumin in g/day; crystalloid preparations in mL/day (estimates per 1 mL). 95% CI = 95% confidence interval (Wald).

Variable	Estimate	95% CI	Std. Error	*p* Value
**(Intercept)**	20.7	17.8 to 23.5	1.45	<0.001
**Gelatin (mL/kg/day)**	−0.029	−0.067 to 0.008	0.019	0.124
**Albumin (g/day)**	−0.126	−0.215 to −0.038	0.045	0.006
**Crystalloids (mL/kg/day)**	−0.25	−0.465 to −0.036	0.109	0.023
**Balanced crystalloid (ELO-MEL) (mL/day)**	0.003	0.0004 to 0.0056	0.0013	0.025
**NaCl (mL/day)**	0.0088	0.0018 to 0.0159	0.0036	0.014
**Ringer Lactate (mL/day)**	0.0075	0.0034 to 0.0116	0.0021	<0.001
**Electrolyte-glucose solution (Perflux) mL/day)**	0.0073	0.0016 to 0.0131	0.003	0.014

## Data Availability

Data will be made accessible upon request.

## Supplementary Materials

The following supporting information can be downloaded at https://www.mdpi.com/article/10.3390/jcm15218598/s1, Supplementary Table S1: Number of patients and observations contributing to each analysis; Supplementary Table S2: Availability of daily KDIGO staging and reasons for missing data (*n* = 220 trauma patients); Supplementary Table S3: Timing of AKI and renal outcomes; Supplementary Table S4: Effect of gelatin in the adjusted models: complete-case analysis vs. multiple imputation of missing covariates; Supplementary Table S5a–c: Competing-risk analysis: study population and events (ICU days 2–7), Cause-specific hazard ratios for the gelatin dose of the previous day (per 1 mL/kg), Fine–Gray model for new-onset AKI with death and ICU discharge as competing events; Supplementary Table S6: Additional sensitivity analyses for the association between gelatin and kidney injury; Supplementary Table S7: Patients included in and excluded from the cluster analysis, and cluster stability; Supplementary Table S8: Spearman rank correlation between gelatin dose (mL/kg/day) and clinical parameters on ICU days 1–7, adjusted for multiple testing; Supplementary Figure S1: Patient flow and analysis samples; Supplementary Figure S2: Cumulative incidence of new-onset AKI, death and ICU discharge by day-1 gelatin dose (median split).

## Abbreviations

The following abbreviations are used in this manuscript:

AKIN	Acute Kidney Injury Network
BE	Base Excess
cmprsk	Competing Risks
CNS	Central Nervous System
CRP	C-Reactive Protein
ED	Emergency Department
EK	Ethikkommission (Ethics Committee)
EMA	European Medicines Agency
ER	Emergency Room
GFR	Glomerular Filtration Rate
ggplot2	Grammar of Graphics Plotting Package (R library)
HES	Hydroxyethyl Starch
ICU	Intensive Care Unit
IQR	Interquartile Range
ISS	Injury Severity Score
KDIGO	Kidney Disease: Improving Global Outcomes
lme4	Linear Mixed-Effects Models using Eigen and S4
MCF	Maximum Clot Firmness
mice	Multivariate Imputation by Chained Equations
OR	Operating Room
PAM	partitioning around medoids
R	Programming Language “R”
ROTEM	Rotational Thromboelastometry
RRT	Renal Replacement Therapy
SAPS 3	Simplified Acute Physiology Score 3
TASH	Trauma-Associated Severe Hemorrhage
