# Supplementary material for: Association Between Succinylated Gelatin Exposure and Acute Kidney Injury in Critically Ill Trauma Patients: A Retrospective Five-Year Cohort Study
Source: J Clin Med. 2026 Nov 4;15(21):8598. doi: 10.3390/jcm15218598 (PMC13764186; doi:10.3390/jcm15218598)
Supplement: Supplementary file 1 [file jcm-15-08598-s001.zip › jcm-4602771-supplementary.pdf]

Supplementary Material

No independent association between gelatin and kidney injury in critically ill intensive care patients: Evidence from a five-year cohort study

Supplementary Table S1. Number of patients and observations contributing to each analysis

Analysis	Patients	Observations (patient-days)
Baseline characteristics (Table 1)	215	–
Model A (unadjusted)	207	1,047
Models B and C	199	970
Model D	199	969
Model E (patient level)	199	–
Model B without creatinine / with previous-day gelatin	202 / 173	1,003 / 795
Multiple imputation (Models B–D)	207	1,047
Competing-risk analysis (cause-specific Cox) / Fine–Gray	196 / 176	822 / –
Model B / Model E excluding AKI at ICU admission	175 / 175	880 / –
KDIGO ≥ 1 / KDIGO 3 (patient level)	199 / 203	–
Inverse-probability weighting: KDIGO ≥ 2 / new-onset AKI	208 / 192	–
Cluster analysis	173	–
FIBTEM MCF model	145	238
Daily correlations gelatin vs. KDIGO, ICU days 1–7	207, 179, 158, 144, 129, 119, 111	–

The number of patients per day in the correlation analyses decreases over time because of ICU discharge, death and RRT censoring (Supplementary Table S2).

Supplementary Table S2. Availability of daily KDIGO staging and reasons for missing data (n = 220 trauma patients)

ICU day	KDIGO available	Died	Censored (RRT)	Discharged from ICU	Not documented
1	214	0	3	0	3
2	182	3	9	2	24
3	159	13	10	22	16
4	144	19	9	35	13
5	129	22	10	49	10
6	119	24	13	58	6
7	111	26	13	62	8

Observations after initiation of renal replacement therapy (RRT) were censored, because KDIGO staging is no longer interpretable under RRT.

Supplementary Table S3. Timing of AKI and renal outcomes

	No KDIGO ≥ 2	KDIGO ≥ 2 on ICU day 1	KDIGO ≥ 2 first on days 2–7
No AKI at ICU admission	167	4	17
AKI at ICU admission	12	11	4

Maximum KDIGO stage during ICU days 1–7 (n = 215): stage 0, 142; stage 1, 37; stage 2, 14; stage 3, 22.

Outcome	Total	No AKI (KDIGO < 2)	AKI (KDIGO ≥ 2)	p
RRT within ICU days 1-7	15/215 (7%)	2/179 (1.1%)	13/36 (36.1%)	< 0.001
Urine output < 0.5 mL/kg/h >= 6 h (any day)	36/177 (20.3%)	21/147 (14.3%)	15/30 (50%)	< 0.001
ICU mortality	28/215 (13%)	18/179 (10.1%)	10/36 (27.8%)	0.011
30-day mortality	29/215 (13.5%)	19/179 (10.6%)	10/36 (27.8%)	0.013
In-hospital mortality	33/215 (15.3%)	21/179 (11.7%)	12/36 (33.3%)	0.004

RRT = renal replacement therapy, counted from ICU admission. Urine-output criterion: documented urine output < 0.5 mL/kg/h for ≥ 6 h on any ICU day (data available for 177 patients). p: Fisher's exact test.

Supplementary Table S4. Effect of gelatin in the adjusted models: complete-case analysis vs. multiple imputation of missing covariates

Model	Effect measure	Complete case (95% CI)	p	Multiple imputation (95% CI)	p
B: KDIGO (continuous), adjusted	β	-0.0002 (-0.0020 to 0.0016)	0.840	-0.0006 (-0.0024 to 0.0011)	0.474
C: Model B + chronic kidney disease	β	-0.0001 (-0.0019 to 0.0017)	0.887	-0.0006 (-0.0024 to 0.0012)	0.515
D: Model B + sepsis	β	-0.0002 (-0.0020 to 0.0016)	0.795	-0.0006 (-0.0024 to 0.0011)	0.476
E: KDIGO ≥ 2 on any ICU day (logistic), adjusted as B	OR	1.039 (0.995 to 1.086)	0.086	1.034 (0.994 to 1.075)	0.099
Model B without creatinine	β	-0.0010 (-0.0030 to 0.0009)	0.304	-0.0012 (-0.0031 to 0.0007)	0.226
Model B, gelatin of previous day	β	-0.0006 (-0.0023 to 0.0011)	0.466	-0.0006 (-0.0022 to 0.0010)	0.441

Per 1 mL/kg/day of gelatin (Model E: mean daily dose, patient-level logistic regression). β = change in KDIGO stage. Multiple imputation by chained equations (30 imputations, predictive mean matching), estimates pooled with Rubin's rules. Adjustment as in Table 2.

Supplementary Table S5a. Competing-risk analysis: study population and events (ICU days 2–7)

	n
Trauma cohort	220
No KDIGO data	2
AKI on ICU day 1 or RRT before/on day 1 (excluded)	18
Death or ICU discharge on day 1 (not at risk)	4
Analysis cohort	196
New-onset AKI (KDIGO ≥ 2 or RRT)	23
Competing event: death	12
Competing event: ICU discharge	67
Censored (day 7 or last observation)	94

Supplementary Table S5b. Cause-specific hazard ratios for the gelatin dose of the previous day (per 1 mL/kg)

Event	Model	HR	95% CI	p	Events
New-onset AKI	unadjusted	1.002	0.987 to 1.018	0.798	23
New-onset AKI	adjusted for SAPS 3 and CKD	0.997	0.976 to 1.019	0.779	20
Death	unadjusted	1.009	0.987 to 1.031	0.439	12
Death	adjusted for SAPS 3 and CKD	1.009	0.975 to 1.045	0.593	12
ICU discharge	unadjusted	0.911	0.881 to 0.941	< 0.001	67
ICU discharge	adjusted for SAPS 3 and CKD	0.919	0.887 to 0.952	< 0.001	62

Cox proportional hazards models with the gelatin dose of the previous day as time-dependent covariate (counting-process format), robust standard errors clustered by patient. CKD = chronic kidney disease.

Supplementary Table S5c. Fine–Gray model for new-onset AKI with death and ICU discharge as competing events

Variable	Subdistribution HR	95% CI	p
Gelatin ICU day 1 (per 1 mL/kg)	1.001	0.985 to 1.018	0.870
SAPS 3 (per 10 points)	1.623	1.275 to 2.068	< 0.001
Chronic kidney disease	0.655	0.088 to 4.895	0.680

Gray's test comparing cumulative incidence by day-1 gelatin dose (median split): new-onset AKI p = 0.073, death p = 0.556, ICU discharge p < 0.001.

Supplementary Table S6. Additional sensitivity analyses for the association between gelatin and kidney injury

Analysis	Estimate (95% CI)	p	n / events
Model A, patients without AKI at ICU admission	β -0.0012 (-0.0031 to 0.0007)	0.214	183 patients
Model B, patients without AKI at ICU admission	β -0.0011 (-0.0029 to 0.0007)	0.235	175 patients
Model E, patients without AKI at ICU admission	OR 1.051 (0.971 to 1.138)	0.219	175 / 20
KDIGO >= 1 on any ICU day (adjusted as Model B)	OR 1.034 (0.992 to 1.077)	0.111	199 / 70
KDIGO >= 2 on any ICU day (Model E) (adjusted as Model B)	OR 1.039 (0.995 to 1.086)	0.086	199 / 33
KDIGO 3 on any ICU day (adjusted for creatinine and AKI at admission)	OR 1.037 (1.001 to 1.073)	0.042	203 / 21
New-onset AKI ~ cumulative gelatin up to previous day, adjusted for SAPS 3 and CKD	HR 0.997 (0.980 to 1.014)	0.722	196 / 20
KDIGO stage ~ cumulative gelatin to date (linear mixed model, adjusted as Model B)	β 0.0021 (0.0007 to 0.0034)	0.003	199 patients
RRT initiation on ICU days 2–7 ~ gelatin of previous day (cause-specific Cox), unadjusted	HR 1.009 (0.978 to 1.041)	0.582	206 / 10
RRT initiation on ICU days 2–7 ~ gelatin of previous day, adjusted for SAPS 3	HR 0.978 (0.949 to 1.007)	0.140	206 / 10
Gelatin ≥ 10 mL/kg on ICU day 1: KDIGO >= 2 during ICU days 1-7, unweighted	OR 2.42 (0.70 to 8.39)	0.164	208 / 34
Gelatin ≥ 10 mL/kg on ICU day 1: KDIGO >= 2 during ICU days 1-7, IPW	OR 1.75 (0.31 to 9.71)	0.526	208 / 34
Gelatin ≥ 10 mL/kg on ICU day 1: New-onset AKI (days 2-7), unweighted	OR 4.68 (0.61 to 36.15)	0.139	192 / 22
Gelatin ≥ 10 mL/kg on ICU day 1: New-onset AKI (days 2-7), IPW	OR 1.28 (0.16 to 10.13)	0.814	192 / 22

Gelatin per 1 mL/kg/day (β: change in KDIGO stage; OR: patient-level logistic regression, mean daily dose) unless stated otherwise. An ordinal mixed model for daily KDIGO stage could not be estimated reliably because KDIGO stage rarely changes within patients.

Tertile of gelatin dose on ICU day 1	Range (mL/kg)	Patients	New-onset AKI
T1 (lowest)	0-18.8	65	4 (6.2%)
T2	19-38.9	65	10 (15.4%)
T3 (highest)	40-107.7	62	8 (12.9%)

Competing-risk cohort (n = 192 with day-1 dose); p for trend adjusted for SAPS 3 = 0.465.

Subgroup (Model B with interaction term)	n (subgroup)	β outside subgroup (95% CI)	β in subgroup (95% CI)	p interaction
Age >= 65 years	58	-0.0000 (-0.0019 to 0.0018)	-0.0008 (-0.0047 to 0.0030)	0.677
SAPS 3 >= median	100	-0.0006 (-0.0031 to 0.0018)	-0.0014 (-0.0036 to 0.0008)	0.575
Lactate (ER) >= 2 mmol/L (18 mg/dL)	93	0.0029 (-0.0001 to 0.0058)	0.0001 (-0.0022 to 0.0025)	0.084
Systolic BP (ER) < 90 mmHg	28	-0.0005 (-0.0027 to 0.0016)	-0.0004 (-0.0033 to 0.0026)	0.929
Creatinine at ICU admission > 1.2 mg/dL	33	0.0022 (-0.0001 to 0.0045)	-0.0136 (-0.0188 to -0.0084)	< 0.001
Chronic kidney disease	14	-0.0001 (-0.0019 to 0.0017)	-0.0061 (-0.0189 to 0.0067)	0.357
Fluid overload > 10% at any time	56	0.0009 (-0.0015 to 0.0032)	-0.0010 (-0.0032 to 0.0012)	0.179

β per 1 mL/kg/day of gelatin.

Covariate	SMD before weighting	SMD after weighting
Age	-0.11	-0.03
Female sex	-0.16	0.05
SAPS 3	0.52	0.04
ISS	0.39	0.04
Chronic kidney disease	-0.04	0.01
AKI at ICU admission	-0.18	-0.04
Creatinine at ICU admission	0.4	0.21
Lactate (ER)	0.42	0.12
Systolic BP (ER)	-0.65	-0.2
Base excess at ICU admission	-0.17	0.16

Covariate	SMD before weighting	SMD after weighting
Polytrauma	0.14	0.01

Balance of the propensity-score model (standardized mean differences, SMD). Exposure: gelatin ≥ 10 mL/kg on ICU day 1 (172 of 208 patients). Stabilized weights truncated at the 1st and 99th percentiles; missing covariate values replaced by the median with missing-indicator variables.

Supplementary Table S7. Patients included in and excluded from the cluster analysis, and cluster stability

Variable	Included (n = 176 records)	Not included (n = 39)	p
Age	51 (34.75-63)	55 (33.5-72.5)	0.225
SAPS 3	48 (37-59)	44 (29.5-68.5)	0.667
ISS	25 (17-34.25)	13 (9-29)	0.003
ICU length of stay (days)	9 (4.5-15.5)	1 (1-1.5)	< 0.001
Gelatin ICU day 1 (mL/kg)	28.45 (15.21-46.13)	14.72 (6.5-27.24)	0.002
Days with KDIGO staging	7 (4-7)	1 (1-1)	< 0.001
KDIGO >= 2 (any day)	28/176 (15.9%)	8/39 (20.5%)	0.482
ICU mortality	16/176 (9.1%)	12/39 (30.8%)	< 0.001
AKI at ICU admission	16/176 (9.1%)	11/39 (28.2%)	0.003

Median (IQR) or n/N (%). 36 of the 39 patients not included had no lactate measurement after ICU day 1. The 176 records correspond to 173 patients in the cluster analysis.

Cluster	Patients	Mean Jaccard coefficient (200 bootstrap resamples)	Dissolved in resamples
1	5	0.36	142/200
2	142	0.92	0/200
3	26	0.65	76/200

Partitioning around medoids on a weighted Gower distance, k = 3 (mean silhouette width 0.472 for k = 3 vs. 0.468 for k = 2). Jaccard coefficients ≥ 0.75 indicate stable clusters, 0.6–0.75 patterns, < 0.5 unstable clusters.

Supplementary Table S8. Spearman rank correlation between gelatin dose (mL/kg/day) and clinical parameters on ICU days 1–7, adjusted for multiple testing

Variable	Day 1	Day 2	Day 3	Day 4	Day 5	Day 6	Day 7
KDIGO	0.05	0.15	0.05	-0.03	0.09	0.14	0.11
AKIN	0.03	0.15	0.07	0.06	0.14	0.19	0.19
Diastolic BP	-0.17	-0.19	-0.07	-0.03	-0.13	-0.03	-0.07
Systolic BP	-0.19*	-0.10	-0.07	-0.14	-0.19	-0.23	-0.16
Lactate	0.37***	0.28**	0.11	-0.00	0.17	0.15	-0.01
Creatinine	0.05	0.06	0.11	0.01	0.10	0.13	0.09
Fluid Balance	0.37***	0.43***	0.22*	0.11	0.16	0.25*	0.22
Na+	0.39***	0.18	0.14	0.10	0.10	0.22	0.07
Cl-	0.22*	0.17	0.14	0.04	0.06	0.25*	0.04
CRP	-0.30***	-0.03	0.11	-0.15	-0.00	0.26*	0.21
Fibrinogen	-0.44***	-0.25**	-0.06	-0.27**	-0.17	-0.02	0.03
Urine Output	0.14	0.17	0.06	0.16	0.10	-0.09	0.05
Antithrombin	-0.58***	-0.49***	-0.35***	-0.31**	-0.33**	-0.39***	-0.25*
Crystalloids	0.13	0.22*	0.17	0.12	0.17	0.05	0.13
Base Excess	-0.12	-0.07	-0.20	0.00	-0.09	-0.14	-0.14
Hemoglobin	-0.11	-0.05	0.00	0.16	-0.12	-0.05	0.05
Renal SOFA	0.14	0.12	0.07	-0.08	0.01	0.13	0.01
MAP	-0.30***	-0.36**	-0.17	-0.09	-0.24	-0.21	-0.20

** $p < 0.05$, ** $p < 0.01$, *** $p < 0.001$ after Benjamini–Hochberg adjustment across all 126 tests. Day-1 values were computed from the ICU time points 0–6 h, 6–12 h and 12–24 h. Descriptive analysis, not adjusted for confounding.*

Supplementary Figure S1. Patient flow and analysis samples

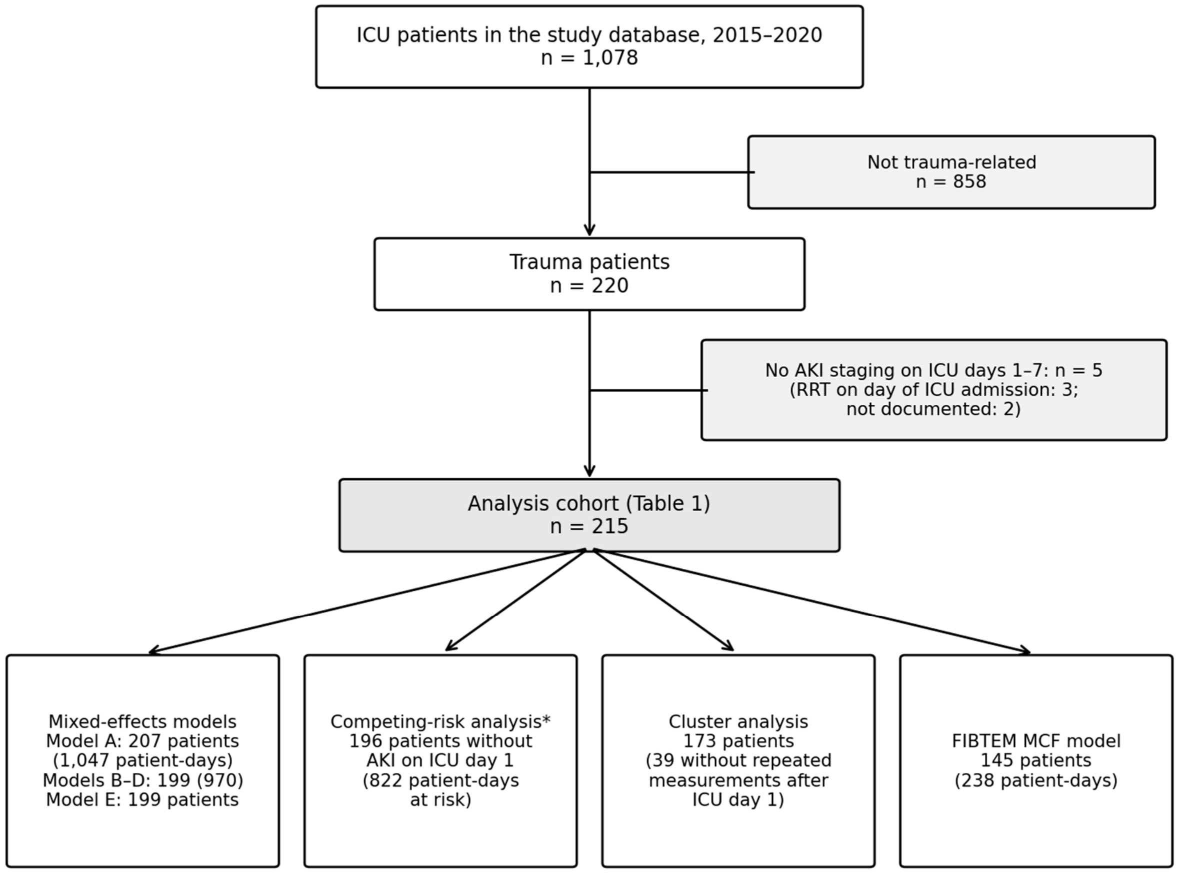

**Based on all 220 trauma patients; patients with AKI on ICU day 1 or death/discharge on ICU day 1 excluded.*

Supplementary Figure S2. Cumulative incidence of new-onset AKI, death and ICU discharge by day-1 gelatin dose (median split)

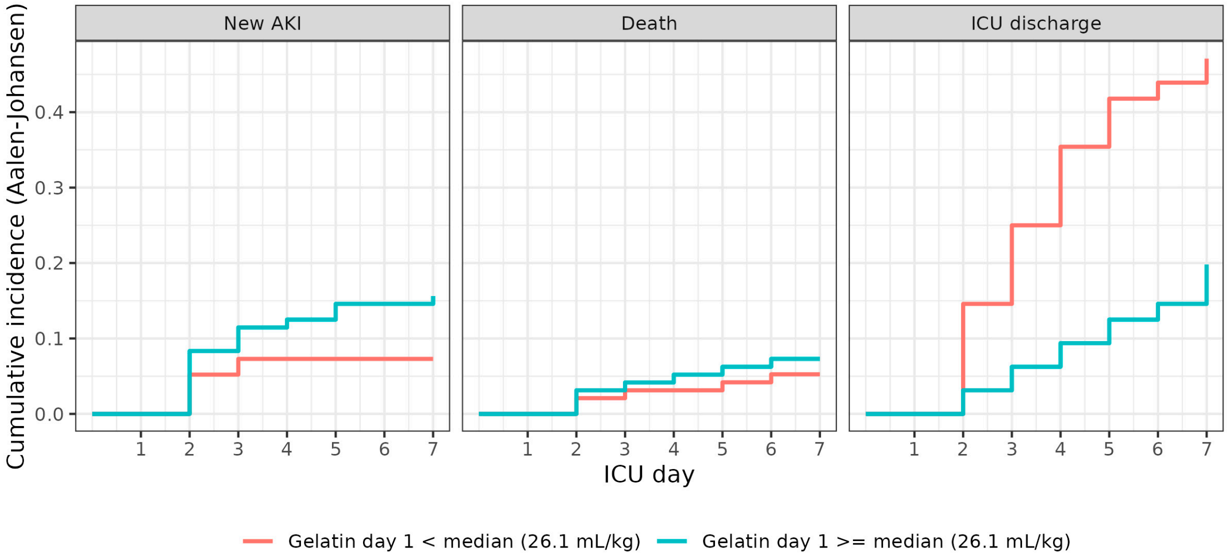

Aalen–Johansen estimates. Patients with AKI on ICU day 1 were excluded.
